# Society Meetings

**Published:** 1910-10

**Authors:** 


### SOCIETY MEETINGS

The Seventh International Tuberculosis Congress will be held in Rome, September 24-30, 1911, official announcement of which was made September 15, 1910, from the American headquarters by the National Association for the Study and Prevention of Tuberculosis. This gathering, which meets every three years, and was last held in Washington, D. C., in 1908, will be under the direct patronage of the King and Queen of Italy. The Secretary-General is Professor Vittorio Ascoli, and the President Professor Guido Baccelli.

It is expected that an American Committee of One Hundred will be appointed as the official body representing the United States. Estimating on the present rate of increase, the National Association for the Study and Prevention of Tuberculosis announces that the American Committee will be able to give a most flattering report at the Congress. They will be able to announce that the number of tuberculosis agencies in this country has tripled in the three years since the last International Congress, and that more than twice as much money is being spent in the fight against consumption by private societies and institutions, and also that the appropriations for tuberculosis work by federal, state, municipal, and county authorities have quadrupled.

The Congress at Rome will be in three sections, that on etiology and causes of tuberculosis; on pathology and therapeutics, both medical and surgical; and on the social defence against tuberculosis. The names of the presidents of these sections will be announced in the near future.

---

The forty-third annual meeting of the Medical Association of Central New York, will be held at Syracuse, October 19 and 20, 1911, under the presidency of Dr. F. W. Sears, of that city. These meetings are always of interest and this one promises to be unusually so. The secretary is Dr. C. R. Greenleaf, of Hornell, who may be addressed in regard to the program. The Fifth District Branch of the Medical Society of the State of New York, will meet in joint session with the Medical Association of Central New York. Dr. William H. Welch, of Baltimore, president of the American Medical Association will address the joint meeting on one of the days mentioned.

---

The next annual conference of the Health Officers' Association of the State of New York will be held at Buffalo, during Wednesday, Thursday, and Friday, November 16, 17, and 18, 1910. Dr. Francis E. Fronczak, health commissioner, will have charge of the arrangements for the meeting.

---

The fifth annual meeting of the Eighth District Branch of the Medical Society of the State of New York was held in the Lectuie Room of the Buffalo Historical Society, Public Library building, at Buffalo, September 27 and 28, 1910.

The following program was observed: Tuesday, September 27—afternoon session, 2 P. M.—president's address, Edward Munson, Medina; Symposium—Poliomyelitis, Etiology and Epidemiology, L. Kaufmann, Buffalo; Symptomatology, Irving M. Snow, Buffalo; Diagnosis, James W. Putnam, Buffalo; Sequelæ, Prescott Le Breton, Buffalo.

7 P. M.—Subscription dinner and entertainment at the University Club of Buffalo, corner Delaware Avenue and Allen Street.

Wednesday, September 28—morning session, 9.30; business meeting, 10 A. M.—Latent Sinusitis, George F. Cott, Buffalo; Experience with Eclampsia, M. P. Messinger, Oakfield; Gun Shot Wounds of the Abdomen, Thew Wright, Buffalo; Some

Old Truths about Infant Feeding Worth Repeating, Carl G. Leo-Wolf, Niagara Falls; *x*-Ray Diagnosis, Leonard Reu, Buffalo.

Afternoon session—What is the Conservative Treatment of Prostatic Cases? James A. Gardner, Buffalo; Intestinal Obstruction, William D. Johnson, Batavia; Meningitis, Floyd S. Crego, Buffalo; Goitre, G. W. Cottis, Batavia; Prolapse of Uterus and Bladder, Earl P. Lothrop, Buffalo.

The Officers were: president, Edward Munson, Medina; first vice-president, T. H. McKee, Buffalo; second vice-president, Henry A. Eastman, Jamestown; secretary, Carl Tompkins, 150 Allen Street, Buffalo; treasurer, C. A. Wall, Buffalo.

